# Single-cell immune profiling reveals distinct immune response in asymptomatic COVID-19 patients

**DOI:** 10.1038/s41392-021-00753-7

**Published:** 2021-09-16

**Authors:** Xiang-Na Zhao, Yue You, Xiao-Ming Cui, Hui-Xia Gao, Guo-Lin Wang, Sheng-Bo Zhang, Lin Yao, Li-Jun Duan, Ka-Li Zhu, Yu-Ling Wang, Li Li, Jian-Hua Lu, Hai-Bin Wang, Jing-Fang Fan, Huan-Wei Zheng, Er-Hei Dai, Lu-Yi Tian, Mai-Juan Ma

**Affiliations:** 1 Center for Disease Control and Prevention of Chinese People’s Liberation Army, Beijing, China; 2 The Walter and Eliza Hall Institute of Medical Research, Parkville, Vic Australia; 3 Department of Medical Biology, The University of Melbourne, Parkville, Vic Australia; 4 State Key Laboratory of Pathogen and Biosecurity, Beijing Institute of Microbiology and Epidemiology, Beijing, China; 5 The Fifth Hospital of Shijiazhuang, Hebei Medical University, Shijiazhuang, China

**Keywords:** Infectious diseases, Infection, Infectious diseases

## Abstract

While some individuals infected by severe acute respiratory syndrome coronavirus 2 (SARS-CoV-2) present mild-to-severe disease, many SARS-CoV-2-infected individuals are asymptomatic. We sought to identify the distinction of immune response between asymptomatic and moderate patients. We performed single-cell transcriptome and T-cell/B-cell receptor (TCR/BCR) sequencing in 37 longitudinal collected peripheral blood mononuclear cell samples from asymptomatic, moderate, and severe patients with healthy controls. Asymptomatic patients displayed increased CD56^bri^CD16^−^ natural killer (NK) cells and upregulation of interferon-gamma in effector CD4^+^ and CD8^+^ T cells and NK cells. They showed more robust TCR clonal expansion, especially in effector CD4^+^ T cells, but lack strong BCR clonal expansion compared to moderate patients. Moreover, asymptomatic patients have lower interferon-stimulated genes (ISGs) expression in general but large interpatient variability, whereas moderate patients showed various magnitude and temporal dynamics of the ISGs expression across multiple cell populations but lower than a patient with severe disease. Our data provide evidence of different immune signatures to SARS-CoV-2 in asymptomatic infections.

## Introduction

Severe acute respiratory syndrome coronavirus 2 (SARS-CoV-2), the causative agent of the coronavirus disease 2019 (COVID-19), has rapidly caused a worldwide pandemic with ever-increasing cases and COVID-19-related deaths.^1^ COVID-19 patient exhibit a broad spectrum of clinical manifestations, ranging from mild or even asymptomatic infection to severe disease or death.^2^ Therefore, understanding the host immune response involved in the disease course is of supreme importance to developing effective therapies.

In severe COVID-19 patients, hyper-inflammation responses referred to as cytokine storm^3,4^ and lymphopenia^5,6^ have been considered risk factors associated with the detrimental progression of COVID-19 patients. Elevated pro-inflammatory cytokines (e.g., IL-1β, IL-6, and TNF-α) and inflammatory monocytes and neutrophils, and a sharp decrease in lymphocytes have also been reported in severe patients.^3,5–12^ Further, single-cell RNA sequencing (scRNA-seq) studies in peripheral blood mononuclear cells (PBMCs) or bronchoalveolar lavages of moderate and severe patients have revealed that moderate disease was associated with more protective T cell-dependent response, with exacerbated systemic inflammation and less effect T cells in severe disease.^9,13–17^ Longitudinal immune responses of moderate and severe COVID-19 patients have been analyzed by flow cytometry,^10^ while unbiased longitudinal single-cell transcriptome profiling is still missing. On the other hand, the contribution of asymptomatic individuals to the transmission of SARS-CoV-2 raises a significant public health concern.^18^ Despite the clinical and immunological assessment of asymptomatic individuals,^19^ transcriptome profiles of asymptomatic individuals are lacking, which might help us understand the nature of the asymptomatic COVID-19 disease.

To explore characteristics that might lead to immunopathology in asymptomatic and moderate COVID-19, we performed scRNA-seq together with single-cell V(D)J sequencing using longitudinal PBMCs from 16 hospitalized COVID-19 patients and three healthy controls (HCs) to identify immunological profiles between distinct immune phenotype and disease severity.

## Results

### Single-cell transcriptomes profiling of PBMCs from COVID-19 patients

**Fig. 1:**
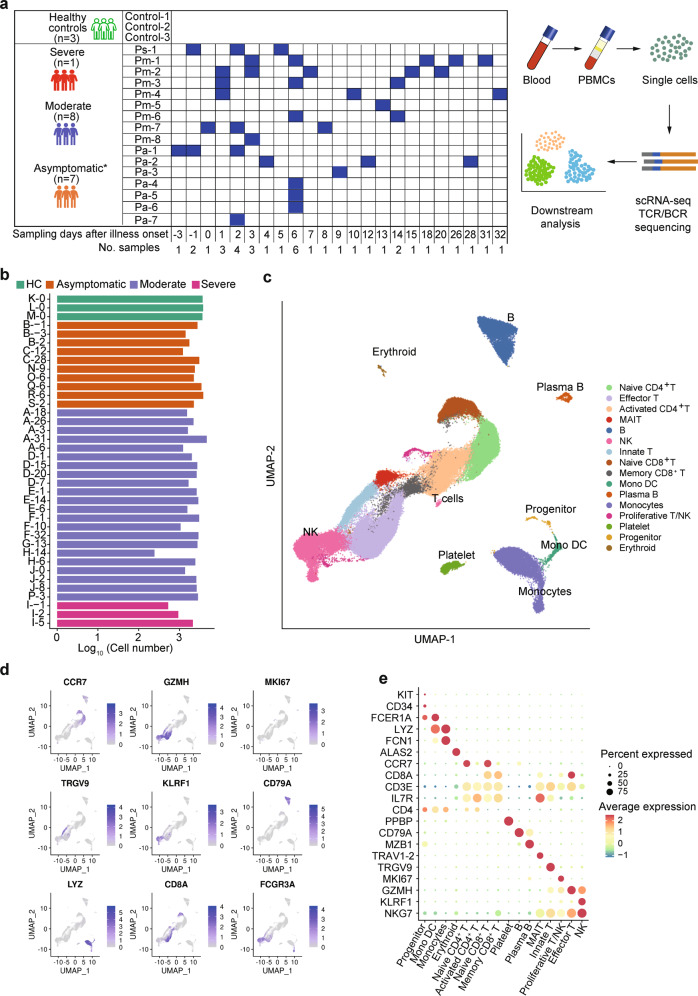
Single-cell RNA sequencing of peripheral blood cells from COVID-19 patients. **a** Timeline of blood sample collection in the 19 subjects (seven asymptomatic, eight moderate, one severe patient, and three healthy controls) and schematic outline of the study design. The days were recorded based on the time after symptom onset for moderate and severe patients and the first positive RT-PCR test for SARS-CoV-2 for asymptomatic patients. **b** Bar plot shows the log_10_ transformed cell number of each sample for each subject at different time points. Green, orange, blue, and red represent samples collected from healthy controls, asymptomatic, moderate, and severe patients, respectively. The same color palette was used throughout the study. **c** Cell type UMAP representation of all merged samples. Sixteen cell types were identified by cluster gene signatures and color-coded. Each dot represents an individual cell. **d** Canonical cell markers that are used to annotate clusters as represented in the UMAP plot. Colored according to expression levels and legend labeled in log scale. **e** Dot plots of average expression and percentage of expressed cells of marker genes in each labeled cell type

A total of 16 laboratory-confirmed COVID-19 patients by real-time reverse transcription-polymerase chain reaction (rRT-PCR) and three HCs were enrolled. The demographics and clinical features of these subjects are shown in Table S1. Of the 16 patients, seven patients were asymptomatic (Pa), eight presented moderate (Pm) disease, and one exhibited severe (Ps) disease. Their ages ranged from 17 to 62 years old, and 12 of them were male. No significant differences in age were found between patient’s groups. A total of 37 blood samples were collected from 16 patients, and eight of 16 patients provided more than or equal to three blood samples at different time points during hospitalization (Fig. 1a).

**Fig. 2:**
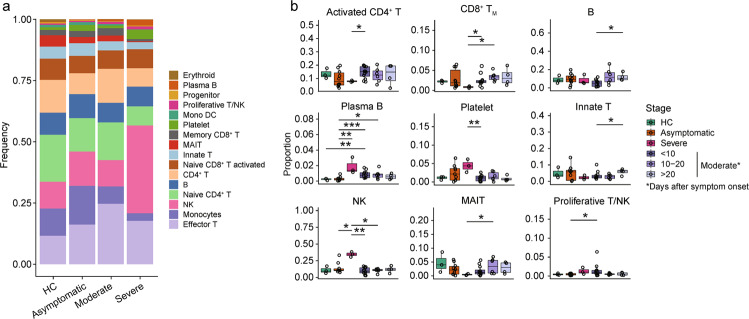
Differences in major cell types compositions across disease conditions. **a** Proportion of cell types in PBMCs of healthy controls (HCs, *n* = 3), moderate (*n* = 8), severe (*n* = 1), and asymptomatic patients (*n* = 7). Colored according to cell type information. **b** Boxplots showing the percentages of each cell type to total cell number per PBMC sample in four disease conditions (HCs, asymptomatic, moderate, and severe) and stages. Boxes are colored according to disease conditions and stages of the moderate condition. The PBMC samples from moderate patients were classified into 3 stages (<10 days, 10–20 days, and >20 days) based on the days after symptom onset. Boxplots indicate the median and interquartile range (IQR); the whiskers represent 1.5 times the IQR. Each circle represents the proportion of each PBMC sample. Two-sided Kruskal–Wallis test was used for analysis, and a *p* value < 0.05 is considered significant. *p < 0.05, **p < 0.01, ***p < 0.001. No asterisk indicates no statistical significance

To explore the immunological changes in patients with asymptomatic, moderate, and severe disease, we analyzed the immune profiles of PBMCs from 16 patients and three HCs by scRNA-seq using the 10× Chromium platform (Fig. 1a). A total of 88,374 cells were included for analysis, including 77,168 cells from 16 patients and 11,206 cells from three HCs. On average, there were 2300 cells for each PBMCs sample (Fig. 1b). We identified 16 major cell types (Fig. 1c–e; Supplementary Fig. 1a–c), including mucosal-associated invariant T (MAIT) cells (*IL17R*^+^), innate T cells (*TRGV9*^+^), effector T cells (*GZMK*^+^), naive CD8^+^ T cells (*CCR7*^+^ *SELL*^+^), memory CD8^+^ T cells (*GPR183*^+^), naive CD4^+^ T cells (*CCR7*^+^ *SELL*^+^), activated CD4^+^ T cells (*IL7R*^+^ *CCR7*^−^), proliferative T/natural killer (NK) cells (*MKI67*^+^), NK cells (*NKG7*^+^), progenitor cells (*CD34*^+^ GATA2^+^), B cells (*CD79A*^+^ *MS4A1*^+^), plasma B cells (*CD38*^+^ *MZB1*^+^), monocytes (CD14^+^ monocytes: *LYZ*^+^; CD16^+^ monocytes: *FCGR3A*^+^), platelet (*PPBP*^+^), monocyte-derived dendritic cells (Mono DC: *CD1C*^+^), and erythroid cells (*ALAS2*^+^). Further comparison of the proportions of 15 cell types among PBMCs that the proportions of T cell subsets were highly heterogeneous among different stages in moderate and asymptomatic patients, including activated CD4^+^ T cells and memory CD8^+^ T cells consistently lower abundance in severe and HCs (Fig. 2a, b; Supplementary Fig. 2a, b). We observed that one severe patient had an increased proportion of NK cells, plasma B cells, and platelets (Fig. 2b). There is no obvious difference in the abundance of major cell types between asymptomatic and moderate patients, except moderate patients had a higher proportion of plasma B cells.

### Immune profiles of T cells and NK cells in COVID-19 patients

**Fig. 3:**
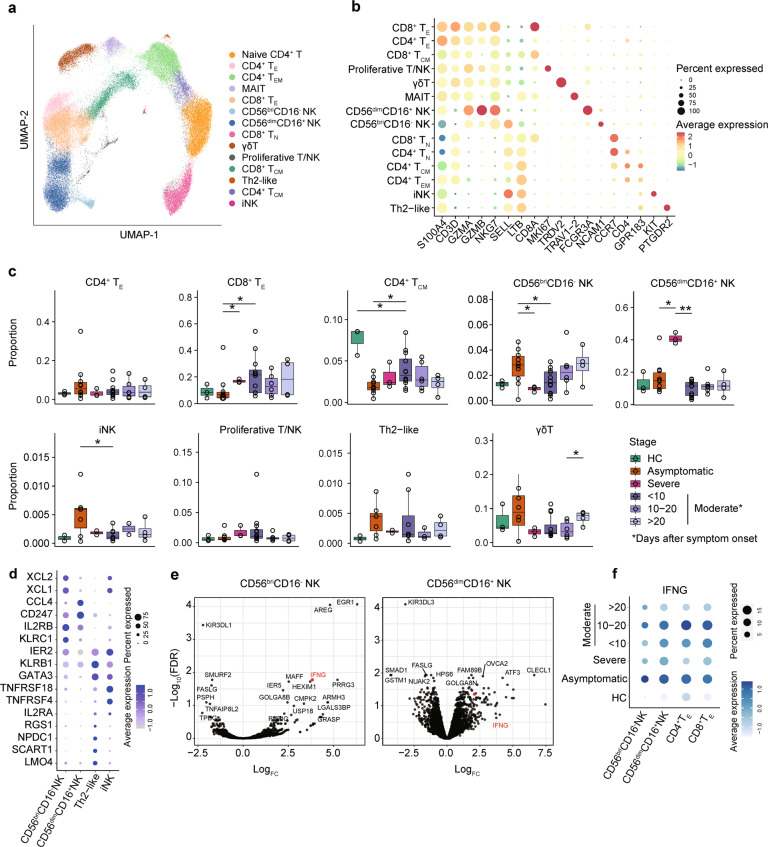
Identification and characterization of the subpopulation of T and innate immune cells in COVID-19 patients. **a** UMAP of T and NK cells by Seurat. Cell types were identified by the marker genes. Each circle represents an individual cell. A total of seven T cell subtypes and seven innate immune cell subtypes were identified and color-coded. **b** Dot plot of average expression and percentage of expressed cells of selected canonical markers in each labeled cell subtype. **c** Boxplot showing the proportions of cell subtypes to the number T and NK cells in each PBMC sample in different disease conditions. Boxes are colored according to disease conditions and stages of the moderate condition. The PBMC samples from moderate patients were classified into 3 stages (<10 days, 10–20 days, and >20 days) based on the days after symptom onset. Boxplots indicate the median and interquartile range (IQR); the whiskers represent 1.5 times the IQR. Each circle represents the proportion of each sample. Two-sided Kruskal–Wallis test was used for analysis, and a *p* value < 0.05 is considered significant. **d** Dot plot showing the average expression and percentage of expressed cells of selected differentially expressed genes (DEGs) between three NK subtypes and Th2-like subset. **e** Volcano plots of top DEGs betwe**e**n asymptomatic and healthy samples for CD56^bri^CD16^−^ (left) and CD56^bri^CD16 ^+^ (right) NK cell subtypes. Genes with a log fold change above 1 and false discovery rate (FDR, Benjamini–Hochberg) less than 0.05 were selected. **f** Dot plot showing the expression of *IFNG* on NK and effect T cells for different disease severity and stages. *p < 0.05, **p < 0.01, ***p < 0.001. HC healthy control. No asterisk indicates no statistical significance

To further characterize the T and NK subsets, we extracted the data from T and NK cells. Fourteen cell subtypes were identified, including four CD4^+^ (naïve–*CCR7*^+^, central memory–*GPR183*^+^ *CCR7*^+^, effector memory–*CCR7*^−^*SELL*^−^*GZMA*^+^, and effector–*GZMA*^+^ *GZMB*^+^) and three CD8^+^ (naïve–*CCR7*^+^, central memory–*GPR183*^+^, and effector–*GZMA*^+^ *GZMB*^+^) T cell subsets, seven innate immune subsets (MAIT–*SLC4A10*^+^ *TRAV1–2*^+^, gamma-delta T (γδT) cells–*TRGV9*^+^ *TRDV2*^+^, immature NK cells, iNK–*KIT*^+^, CD56^bri^CD16^−^ NK cells, CD56^dim^CD16^+ ^NK cells, proliferative T/NK population–*MKI*67^+^), and a previously uncharacterized Th2-like lymphoid population (*CD4*^−^*CD8A*^−^*PTGDR2*^+^) (Fig. 3a, b, Supplementary Fig. 3).

We next compared the abundance of each cell type across disease conditions and stages (Fig. 3c; Supplementary Fig. S4b). We observed a decreased proportion of effector CD8^+^ T cells in asymptomatic patients compared to early-stage (<10 days post symptom onset) of moderate patients, and asymptomatic patients had a decreased proportion of central memory CD4^+^ T compared to early-stage of moderate patients and HCs (Fig. 3c). Of interest, the abundance of CD56^bri^CD16^−^ NK cells were significantly higher in asymptomatic patients than in severe and early stage of moderate patients, and there was an increasing trend in moderate patients over time (Fig. 3c). In contrast, the CD56^dim^CD16^+^ NK, the most abundant NK subset, was substantially enriched in the severe patient. Like CD56^bri^CD16^−^ NK, its precursor iNK also increased in asymptomatic patients compared to moderate patients. These results indicated that asymptomatic patients had distinct T and NK cell responses during infection.

Next, we sought to identify the specific signature of the NK cells with distinct distribution in asymptomatic and severe conditions. We found that CD56^bri^CD16^−^ NK cells have high expression of *XCL1*, *XCL2*, and *IFNG* (Fig. 3d, f), consistent with our knowledge that these cells are efficient cytokine producers.^20^ The Th2-like lymphoid cells were *TCR*^*−*^*CD3*^*−*^*CD4*^*−*^*CD8*^*−*^ but expressed Th2 markers such as *PTGDR2* and *GATA3*; they were classified mainly as Th2-like cells (Supplementary Fig. 5a). We also found that *TNFRSF19* is uniquely expressed in the Th2-like cells in this dataset (Supplementary Fig. 5b). However, *TNFRSF19* is absent in most immune cells according to previous study^21^ and the human lung cell atlas database.^22^ It is highly expressed in epithelial cells such as ciliated cells, which express ACE2 and are considered as entry cells of SARS-CoV-2 (Supplementary Fig. 5c).

To further investigate the difference of transcriptomes for each cell type of T and NK cells across different conditions, we performed systematic differential gene expression (DGE) analysis (Data S1). We found distinct signatures expressed in severe and asymptomatic patient samples in NK cells and effector T cells (Supplementary Fig. 6a–d). *IFNG* was upregulated in CD56^dim^CD16^+^ NK and CD56^bri^CD16^−^ NK cells in asymptomatic conditions compared to HCs (Fig. 3d–f; Supplementary Fig. 6c, d). We also found *IFNG* expression showed stage-specific expression in moderate samples, with the highest expression at 10-20 days after symptom onset.

**Fig. 4:**
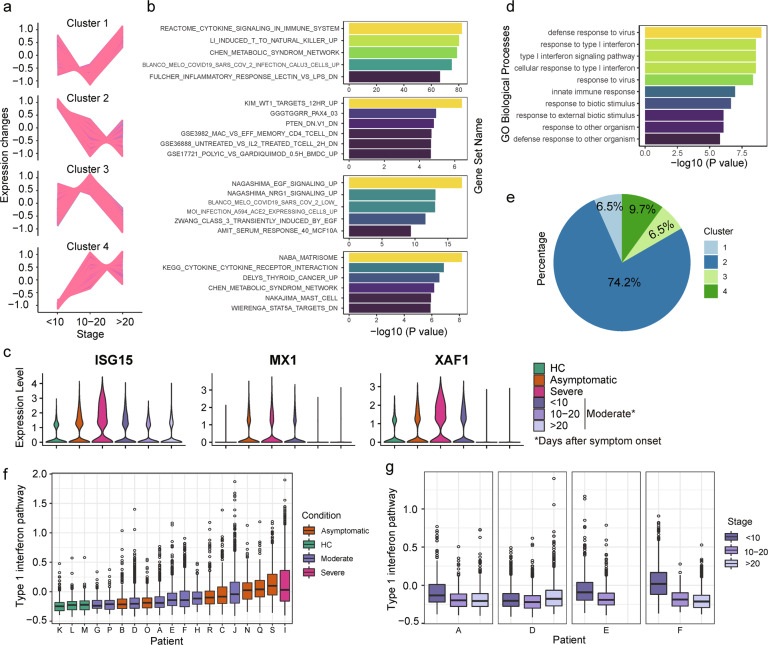
Increased IFN-1 signaling pathway in severe and early stage moderate patients. **a** Gene clusters generated by Mfuzz R package based on genes’ expression pattern along the progression of the disease in moderate patients using NK cells and effector T cells. **b** Top gene sets in gene set enrichment analysis for the gene in each cluster in panel (**a**). Gene sets are colored by log_10_ transformed *p*-values representing genes in the overlap. **c** Violin plots of selected DEGs generated by comparing different conditions. HC, healthy control. **d** Bar plots of top ten enriched GO terms from genes upregulated in severe conditions in CD56^dim^CD16^+^ NK cells. **e** The percentage of IFN-1 related genes found in four clusters in (**a**). **f** Box plots of the IFN-1 pathway activity score calculated across patients on NK and effector T cells. Colors denote disease conditions. **g** Boxplots of the IFN-1 pathway activity score for each moderate patient and summarised at each stage. Colors denote defined disease stages

To further explore the IFN-I pathway activity at the single-cell level, we analyzed the stage-dependent expression patterns and explored related gene sets based on both NK cells and effector T cells of moderate patients. We identified four stage-dependent expression patterns (Fig. 4a) and their related gene sets (Fig. 4b, Data S2). We found upregulation of genes such as EGR1 and NR4A1 (Data S2) in later stages as shown in cluster 3 and is known to be induced by TCR stimulation and enhance T cell functions.^23,24^ Genes expression in cluster 2 decreases with time, and one enriched gene set contains genes downregulated with PTEN knockdown, and PTEN was demonstrated with function promoting type 1 interferon responses and antiviral innate immunity.^25^ Meanwhile, we found that type 1 interferon (IFN-I)-related genes, *ISG15*, *MX1*, and *XAF1*, expressed more severe and moderate patients at the early stage (Fig. 4c), and a group of widely upregulated signatures in disease states are genes in IFN-I signaling pathway, especially in severe condition (Fig. 4d, e, Data S3). We then summarized IFN-I pathway activities per cell and found heterogeneous results among different patients (Fig. 4f, Supplementary Fig. 7a). Apart from HCs who show the stable low activity of IFN-I pathway and cells from severe patients with the highest activity on average, the moderate and asymptotic patients have a wide spectral of IFN-I activity ranging from HCs to severe conditions. In addition to intra-patient variability, the IFN-I pathway activity also varies on time, decreases in later stages in moderate patients, suggesting a recovery of disease (Fig. 4g, Supplementary Fig. 7b). The disease progression stage is hard to define for asymptomatic patients. However, the highly heterogeneous IFN-I activity suggests the asymptomatic patients that we profiled in this study were in different disease stages.

### Clonal expansion in T cells and usage of V(D)J genes COVID-19 patients

**Fig. 5:**
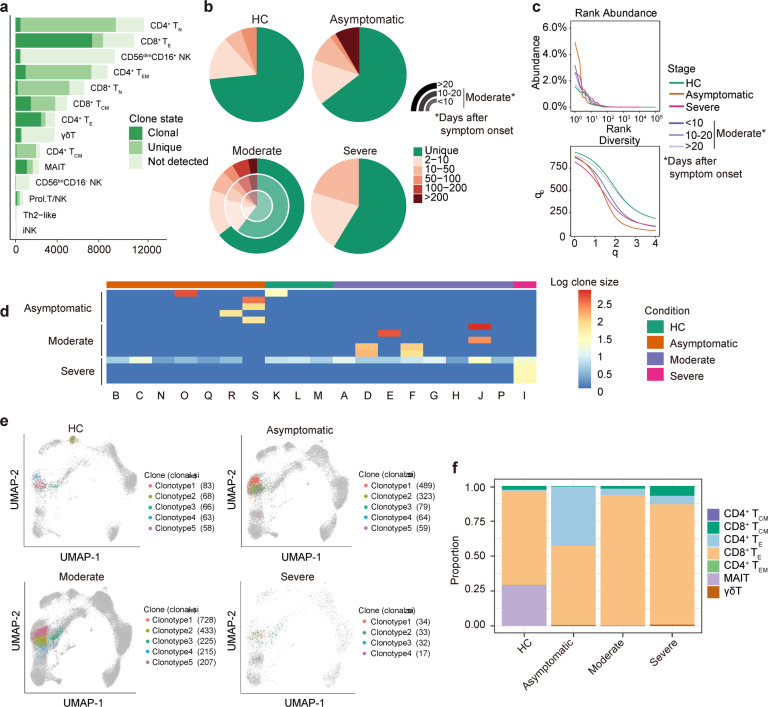
Expanded TCR clones and selective usage of V(D)J genes. **a** Bar plots showing the cells from T and NK cell subtypes whether they have TCR and their TCR clonal status. **b** The percentage of the clonal status of T cells with TCR. The clonal status was defined by clone size in four disease conditions. **c** TCR abundance and diversity on CD4^+^ and CD8^+^ T cells across disease conditions and stages generated by alakazam package. The 95% confidence interval is estimated via bootstrapping (*B* = 200). **d** Heatmap of log_10_-transformed clone sizes of top three abundant clonotypes within each condition (not including healthy controls [HC]) across patients. **e** UMAP of T cells derived from PBMCs for different conditions. Clusters are denoted by colors labeled with TCR clones with the top 5 largest clone sizes in each condition. **f** Proportions of cell types that produce the top five abundant clonotypes within each condition. Color denotes cell types

To evaluate the relationship of clonal expansion among individual T cells and usage of V(D)J genes across different conditions, we analyzed single-cell TCR sequencing data and reconstructed high-quality TCR sequence in 70.5% of the T cells with various degrees of clonal expansion. We observed that both effector CD8^+^ and CD4^+^ T cell subsets displayed more TCR clonal expansion (Fig. 5a; Supplementary Fig. 8a). Both asymptomatic and moderate patients had high clonal expansion compared to the severe patient and HCs (Fig. 5b). We performed a quantitative analysis of TCR clonal abundance and diversity to mitigate the difference in sample size between different conditions. The TCR expansion decreases in moderate patients from early (<10 days) to late-stage (>20 days), indicating recovery of the disease. We found more TCR expansion in asymptomatic patients than moderate patients at the early stage (Fig. 5c). We summarized the distribution of top clone types per condition and found that most of them are COVID-19 specific (Fig. 5d). The clustering of the CDR3 sequence showed a similar sequence enriched in multiple patients, suggest they are reactive to the SARS-CoV-2 virus (Supplementary Fig. 8b). The UMAP visualization of these TCR clones showed that the most abundant clones in asymptomatic and moderate patients have larger clone sizes, consistent with the TCR diversity analysis. It also highlighted the different cell populations in different conditions that enriched for most abundant TCR clones (Fig. 5e). We found that asymptomatic patients have significantly more CD4^+^ effector T cells with the most abundant TCR clones, while most of the abundant TCR clone types were in CD8^+^ effector T cells for moderate and severe patients (Fig. 5f). We then compared the usage of V(D)J genes across disease conditions and disease stages of moderate patients (Supplementary Fig. 8c–e). The preferred *TRBJ* gene in asymptomatic patients was *TRBJ*2–2, *TRBJ*2–1 for moderate patients, and *TRBJ*2–7 for one severe patient (Supplementary Fig. 8c). Collectively, the different patterns of TCR clonal expansion and diversity and the selective usage of V(D)J genes indicated that different immunodominant epitopes may drive the molecular composition of T cell responses and may be associated with SARS-CoV-2-specific infection.

### Features of B cells and expansion and specific rearrangements of V(D)J genes

**Fig. 6:**
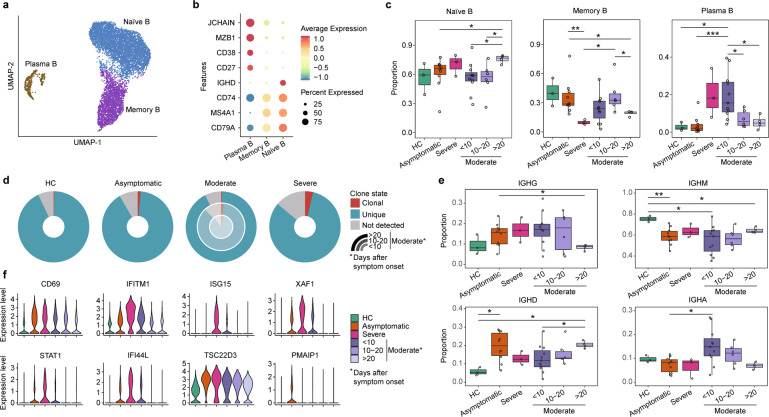
Subpopulation analysis of B cells. **a** UMAP projection of B cells. Each dot represents a single cell, colored according to cell type. **b** Dot plot of canonical cell markers used to annotate clusters in the UMAP plot. **c** Boxplots showing the differences in percentages of each cell type to the number of B cells comparing different disease conditions to healthy controls (HCs). Boxes are colored according to disease conditions and stages of the moderate condition. The PBMC samples from moderate patients were classified into 3 stages (<10 days, 10–20 days, and >20 days) based on the days after symptom onset. Boxplots indicate the median and interquartile range (IQR); the whiskers represent 1.5 times the IQR. Each circle represents the proportion of each sample. Two-sided unpaired Mann–Whitney *U* test was used for analysis, and a *p* value < 0.05 is considered significant. **d** The percentage of clonal status in B cells that have BCR, across disease conditions and stages. **e** Boxplots show the proportion of IgG, IgM, IgD, and IgA, comparing different disease conditions to HCs. Boxes are colored according to disease conditions and stages of the moderate condition. The PBMC samples from moderate patients were classified into 3 stages (<10 days, 10–20 days, and >20 days) based on the days after symptom onset. Boxplots indicate the median and interquartile range (IQR); the whiskers represent 1.5 times the IQR. Each circle represents the proportion of each sample. Two-sided Kruskal–Wallis test was used for analysis, and a *p*-value < 0.05 is considered significant. **f** Violin plots showing the gene expression levels of selected DEGs generated genes in B cells by comparing different conditions. *p < 0.05, **p < 0.01, ***p < 0.001. No asterisk indicates no statistical significance

We extracted single-cell B cells sequencing data and identified three B cell subsets according to the expression of canonical B cell markers, including naïve B cells (*MS4A1*^+^ *IGHD*^+^), memory B cells (*CD27*^+^), and plasma B cells (*MZB1*^+^ *CD38*^+^) (Fig. 6a, b). Moderate patients at an early stage (<10 days after symptom onset) had a higher proportion of plasma B than HCs and asymptomatic patients, and a declining trend of the plasma B proportion was observed over time for the samples of moderate patients (Fig. 6c). We further reconstructed high-quality BCR sequences in more than 80% of the B cells using single-cell BCR sequencing data and found asymptomatic patients displayed less BCR clonal expansion. In contrast, an obvious BCR clonal expansion was observed in moderate patients at the early stage but decreased over time, suggesting humoral immune responses declined at the convalescent stage (Fig. 6d). The severe patient has even more clonal expansion, suggesting a strong humoral immune response (Fig. 6d). Further analysis of the distribution of IgG, IgM, IgD, and IgA at different disease conditions and stages revealed that asymptomatic and moderate patients had lower IgM compared to HCs (Fig. 6e). The IgG was highly variable at the early moderate patient sample and returned to similar levels of HCs at the late stage (>20 days post symptom onset).

We then assessed V(D)J rearrangements of the BCR and analyzed the usage of V(D)J genes across different disease conditions and stages (Supplementary Fig. 9b). We found different skewing of BCR usage in different conditions. The top two paired V–J frequencies in asymptomatic patients were *IGHV*3–23/*IGHJ*4 and *IGLV1–44/IGLJ3*, whereas *IGLV1–44/IGLJ3* and *IGLV2–14/IGLJ2* for moderate patients and *IGLV1–51/IGKJ3* and *IGHV3–33/IGHJ4* for the severe patient (Supplementary Fig. 9b). Collectively, increased B cell clonality from asymptomatic to severe condition and skewed usage of the IGHV and IGKJ genes in different disease conditions suggest that SARS-CoV-2 infection perturbs V(D)J rearrangements of B cells.

To further investigate transcriptomic changes of B cells after SARS-CoV-2 infection, we performed a DGE analysis comparing different conditions in each B cell subsets (Fig. 6f). We found an increased expression of activation maker gene *CD69* in patients, regardless of disease severity, compared to HCs. Like T and NK cells, we observed an increased expression of *IFITM1*, *ISG15*, *XAF1*, *STAT1*, and *IFI44L* involving the IFN-I signaling pathway in the severe and moderate patients, but moderate patients displayed relative low-level expression (Fig. 6f and Supplementary Fig. 9b). By contrast, asymptomatic patients displayed a lack or less expression of these genes.

## Discussion

Although the immune responses to SARS-CoV-2 infection have been studied in patients with moderate and severe disease,^13–17^ the mechanisms under asymptomatic infection of COVID-19 are less studied. In this study, we performed scRNA-seq and V(D)J-sequencing on longitudinal PBMCs from asymptomatic individuals of COVID-19 and systematically compared their immunological characteristics with moderate and severe COVID-19 patients. Our study confirms previously published data on increased type-I interferon (IFN-I) response^26,27^ and the increased plasma B cells and decreased T subsets in severe patient,^10,13–17^ but not for monocytes, which we did not observe might due to the sampling time that only early stage severe samples.^28^ In addition to that, we found that the asymptomatic condition is not just an intermediate state between healthy and moderate but has unique immunological features. Overall, PBMCs in asymptomatic patients had lower IFN-I related gene expression than in severe and early-stage moderate conditions. In contrast, moderate patients at the early stage of infection had an increased IFN-I response, but less than the severe condition and declined over time. However, the heterogeneous IFN-I activity was observed among different patients that 11 patients (4 asymptomatic and 7 moderate patients) had IFN-I activity blow or close to the average level of 0, and the rest of the patients showed more IFN-I activity, reflecting the heterogeneous response of IFN-I induction in patients and even their heterogeneous pathogeneses.^29^ Importantly, a rapid decrease of IFN-I activity is associated with a more rapid recovery of the disease. IFN-I has direct antiviral activity, and their immunopathological role was also previously reported.^30^ The IFN-I response induced the accumulation of pathogenic inflammatory monocytes–macrophages and vascular leakage, leading to death in a BALB/c mice model of acute respiratory distress syndrome (ARDS).^31^ Moreover, a delayed but considerable IFN-I response was proposed that is critical for the development of ARDS and increased lethality during pathogenic human coronavirus infection.^32,33^ Therefore, our data suggest that early moderate IFN-I response might effectively control viral replication and prevent severe COVID-19.

Effector CD4^+^ T cells play a key role in regulating the antiviral inflammatory response and mediating viral clearance through direct cytotoxic effects on virus-infected cells.^34^ Although we observed similar frequencies of effector CD4^+^ T cells among different conditions, the effector CD4^+^ T cells in asymptomatic patients had upregulated *IFNG* compared to other disease conditions (Fig. 3f). Of note, a recent study has shown that the magnitude of T cell responses to SARS-CoV-2 was similar between asymptomatic infections and symptomatic COVID-19 patients,^35^ but SARS-CoV-2-specific T cells produced a higher level of IFN-γ and IL-2 in asymptomatic patient,^35^ which is in line with our study. A previous study also showed that SARS-CoV-2-specific CD4^+^ T cells in severe but not mild COVID-19 displayed low avidity.^36^ In addition, while we observed fewer effector CD8^+^ T cells in asymptomatic patients than moderate and severe patients at the early stage of the infection, the effector CD8^+^ T cells in asymptomatic patients expressed a higher level of *IFNG* but not in severe patient and moderate patients until ten days post-symptoms onset. Because most PBMC samples from asymptomatic patients were collected <10 days after the first positive RT-PCR testing of SARS-CoV-2, we speculated that higher expression of *IFNG* in T cells plays an important role in antiviral infection, especially during the early stage of infection. These data suggest that early activation of effector CD4^+^ and CD8^+^ T cells expressing a higher level of *IFNG* may play an important role in protecting SARS-CoV-2 infection in asymptomatic patients.

Despite the difference in adaptive immune response, we also identified profound differences in innate immune responses in asymptomatic patients compared to moderate and severe patients. Asymptomatic patients had significantly increased CD56^bri^CD16^−^ NK cell fractions than moderate and severe patients at the early stage. We found CD56^bri^CD16^−^ NK subsets had upregulated cytokine-related genes such as *XCL1*, *XCL2*, and *IFNG*, consistent with our knowledge that these regulatory cells act as potent cytokine and chemokine producers. In addition to the difference in abundance, there were significant differences in gene expressions between asymptomatic and moderate conditions, with cytokine-related genes such as *IFNG* upregulated in asymptomatic conditions, but not severe patient and moderate patients until ten days post-symptoms onset. CD56^bri^CD16^−^ NK cells have been linked to virus infection. Infection of influenza A Virus-induced NK cell hyperresponsiveness and cytokine production, particularly in the CD56^bri^CD16^−^ NK subset.^37^ An asymptomatic hemophiliac patient co-infected with HIV/HCV also had increased CD56^bri^CD16^−^ NK cells.^38^ These results suggest that the CD56^bri^CD16^−^ regulatory NK cells may play a critical role in protecting SARS-CoV-2 infection in asymptomatic patients.

TCR and BCR repertoire profiling are important to reflect the disease’s adaptive immune status and develop new therapeutics for infectious disease. Therefore, another central part of our study is to assess the repertoire diversity of TCR and BCR and especially their clustering, enabling us to deduce COVID-19-relevant TCR or BCR signatures. Of note, we found more TCR clonal expansion in asymptomatic patients compared to moderate patients (also highly diverse than severe patient) at the early stage and severe patient, which is in line with previous studies that in patients with a moderate^14,39^ or mild^36,40^ clinical course and but not patients with a severe clinical course, T cell repertoires displayed high clonality. Moreover, the most abundant TCR clones were observed in CD4^+^ effector T cells of asymptomatic patients and CD8^+^ effector T cells of moderate and severe patients. These results indicate that the effective TCR diversification on CD4 or CD8 T cells may contribute the outcome and immune control in COVID-19.

While asymptomatic COVID-19 patients displayed more abundant TCR diversity, on the contrary, they showed less BCR clonal expansion compared to moderate and severe patients. which is in line with the previous studies^28,40,41^ and are likely to be associated with antibody-secreting B cells that produce antibodies to neutralize the infecting pathogen.^41,42^ We found that patients with different conditions shared a common IGLV1/2 and IGLJ2/3 usage pattern and IGHV3 and IGHJ4 usage. Moreover, we found a higher proportion of IGHV3 and IGHJ4 usage in asymptomatic patients. In agreement with our study, IGHV3 and IGHJ4 usage were identified in clusters specific for antibody-positive individuals with COVID-19^40^ and moderate to severe patients.^14^ Notably, a high proportion of IGHV3 and IGHJ4 usage was also observed in HCs.^14^ Previous studies have shown extensive class switching to IgG and IgA subclasses with limited somatic hypermutation.^40,43^ Because our study produced few B cells (~300 cells per sample) and a low percentage of clonal BCR, we could not perform clustering antibody class switching, somatic hypermutation other analyses. Despite our study and other studies that have provided a relationship between BCR repertoire and disease infection, detailed work on BCR repertoire is needed to reveal its role in disease progression in asymptomatic patients.

There are several limitations to this study. One of the major limitations is only one patient with severe COVID-19 and paucity of later samples from the severe patient. These blood samples from patients with severe COVID-19 are challenging to obtain since very few newly diagnosed patients were reported in China when our study was initiated. Although the results from one severe patient are consistent with previous studies, it will be essential to gather such samples further to identify the immune characteristic between asymptomatic and severe COVID-19. Second, because of frequent blood draws of patients during their hospitalization for clinical lab testing, they only provide small volume blood samples, resulting in insufficient PBMCs to detect virus-specific T cells and B cells by flow cytometry to assess their relationship with results of scRNA-seq.

Our data clearly show the immune profiles in asymptomatic COVID-19 patients and highlight the difference of immune response toward disease progression. The specific signatures in asymptomatic patients will increase understanding of COVID-19 disease severity and guide early prediction and therapeutics.

## Materials and methods

### Ethics statement

The study was conducted following the Declaration of Helsinki, and the Institutional Review Board of the Academy of Military Medical Sciences approved the study protocol (IRB number: AF/SC-08/02.46). All patients or their surrogates provided written informed consent.

### Patients

Sixteen patients diagnosed with SARS-CoV-2 infection were enrolled from the Fifth Hospital of Shijiazhuang from March to April 2020. SARS-CoV-2 RNA was detected in the patient’s nasopharyngeal swab or sputum specimens by real-time reverse-transcriptase PCR (RT-PCR) using the SARS-CoV-2 nucleic acid detection kit (Cat No. DA0930-DA0932, DAAN GENE Ltd., Guangzhou, China). Peripheral blood was collected from all patients during hospitalization, and blood draws from patients occurred in concert with usual care and the patient’s willingness to avoid frequent blood sampling and unnecessary personal protective equipment usage. The patients’ demographic, clinical features, laboratory findings, and chest radiographs were collected from their electronic medical records.

According to the diagnostic and treatment guidelines for SARS-CoV-2 issued by the Chinese National Health Committee (Trail Version 7), the disease severity was defined as asymptomatic, moderate, and severe. Asymptomatic infection was defined as an individual who had a positive SARS-CoV-2 by RT-PCR without any associated clinical symptoms in the preceding 14 days and during hospitalization. Moderate was defined according to the following criteria: (i) fever and respiratory symptoms; (ii) radiological signs of pneumonia. Severe was defined if satisfying at least one of the following items: (i) breathing rate ≥30/min; (ii) pulse oximeter oxygen saturation (SpO_2_) ≤ 93% at rest; (iii) ratio of the partial pressure of arterial oxygen (PaO_2_) to a fraction of inspired oxygen (FiO_2_) ≤ 300 mm Hg (1 mm Hg = –0.133 kPa).

### Isolation of PBMCs

PBMCs were isolated from whole blood using density gradient centrifugation with Lymphoprep in SepMate tubes (Stemcell Technologies) in a biosafety level 2 plus facility according to the manufactory’s instruction. Briefly, the blood was centrifuged at 1200 × *g* for 10 min. PBMCs were harvested and washed twice with PBS at 400 × *g* for 10 min. Isolated PBMCs were frozen in cell recovery Media containing 10% DMSO (GIBCO), supplemented with 90% heat-inactivated fetal bovine serum, and stored liquid nitrogen before assays analyses.

### The droplet-based single-cell RNA sequencing

Single-cell suspensions at a density of 1000 cells/μl in PBS plus 0.04% bovine serum albumin were prepared for scRNA-seq using the Chromium Single Cell 5′ Reagent version 2 kit and Chromium Controller (10× Genomics, Pleasanton, CA), aiming for an estimated 4000 cells per library following the manufacturer’s instructions. Briefly, 9000 cells per reaction were loaded for gel bead-in-emulsion (GEM) generation and barcoding. GEM-RT, post-GEM-RT cleanup, and cDNA amplification were performed to isolate and amplify cDNA for library construction. Libraries were constructed using the Chromium Single Cell 5′ Reagent kit (10×Genomics) and Gel Bead Kit, Single Cell V(D)J Enrichment Kit, Human T Cell (1000005) and a Single Cell V(D)J Enrichment Kit, Human B Cell (1000016) according to the manufacturer’s protocol. Library quality and concentration were assessed according to the manufacturer’s instructions. Libraries were sequenced on an Illumina PE150.

### Single-cell RNA-seq data processing

Reads from each sample were processed with Cell Ranger (3.0.1) separately. Human reference genome GRCh38 and genome of SARS-CoV-2 were merged with corresponding GTF files used to annotate genes. The filtered matrices were then delivered into R (3.6.2) for downstream analysis. In order to demultiplex samples pooled into one sequencing run, we applied Souporcell (2.0)^44^ to separate them by individuals. Next, we used the *shared_samples.py* script in Souporcell to identify individuals. The script uses vcf files to compare shared variations when overlapping patients between the two runs and identifies the shared patient.

Quality control was performed using R′s scatter (1.14.6)^45^ to remove cells with: (1) more than three median absolute deviations (MADs) of the log_10_ read counts below the median; (2) more than three MADs of the log_10_ genes detected below the median; and (3) more than three MADs of the genes coming from mitochondria above the median. Size factors were then considered for calculating average counts per feature, and features with average counts above 0 were kept. COVID-19 genes were removed in this step as they are not detected in the data. Afterward, we used Seurat (3.2.0)^46^ for data normalization and to identify highly variable genes.

### Data integration and clustering

*RunFastMNN*^47^ wrapped in Seurat was performed using the top 2000 highly variable genes to integrate data sets from each sample. The first 30 MNN dimension reductions were applied to construct an SNN graph and *FindClusters* with Louvain algorithms using a standard Seurat pipeline. UMAP was also generated with the first 30 MNN dimension reductions to embed the data sets into two dimensions for visualization. Doublets labeled by Souporcell and clusters enriched for doublets (>15%) were removed from further analysis. We removed ribosomal and mitochondrial genes to explore the subtypes of T/NK cells and B cells, then performed the integration and clustering again using the same strategies.

### Cell-type annotation

To annotate each cluster, we used *FindAllMarkers* in Seurat to find marker genes for each cluster and selected immune cells marker genes. SingleR (1.0.5)^48^ was also applied to help interpretation with Monaco Immune Data^49^ (Monaco Immune Cell Data (GSE107011)) used as reference data to annotate the Th2-like cell type. We applied *FindMarkers* in Seurat to compare the innate immune subsets that have distinct distribution in asymptomatic conditions. We searched the expression of ACE2 and TNFRSF19 using the human lung atlas visualization tool (https://cellxgene.cziscience.com/d/krasnow_lab_human_lung_cell_atlas_10x-1.cxg/). The heatmaps that present the proportions of each cell type were generated using the proportions calculated as detailed above and are scaled by row to be plotted on the same color scale.

### DEG analysis

For subtypes of T cells, NK cells, and B cells with >200 cells, we aggregated the counts for cells in each sample and generated pseudobulk samples, following the data analysis workflow specified by a previous study.^50^ Non-protein-coding genes and genes related to sex were removed in the counts before being analyzed by edgeR (3.28.1).^51^ *glmQLFit* and *glmQLFTest* from edgeR were used to find marker genes between each condition. Genes with a log fold change above 1 and FDR (Benjamini-Hochberg) less than 0.05 were selected. Then, genes with logCPM above five were shown in heatmaps in Fig. S6. GO analysis was conducted on upregulated genes using topGO (3.28.1),^52^ and the top 10 enriched GO terms were plotted. Mfuzz package^53^ was applied to cluster gene expression patterns on stages in moderate patients. Cells were aggregated by stages from on CD56^bri^CD16^−^ NK cells, CD56^dim^CD16^+^ NK cells, and CD4/CD8 effector T cells. Genes with more than 25% are missing values are excluded, normalized summed counts are standardized. We clustered the standardized genes into 4 clusters. Genes in each cluster are then used as identifiers to compute overlaps with C2 curated gene sets in MsigDB.^54^ To calculate the percentage of IFN-1 related genes in the clusters, genes from GO: 0034340 were used. The IFN-1 score was calculated on CD56^bri^CD16^−^ NK cells, CD56^dim^CD16^+^ NK cells, and effector T cells using genes involved in the pathway as input, including *IFIT1*, *IFIT2*, *IFIT3*, *IRF9*, *OAS3*, *RSAD2*, *USP18*, *IFI27*, *ISG15*, *MX1*, and *XAF1*. The AddModuleScore function in Seurat was used to calculate the score.

### TCR and BCR analysis

Raw fastq files were processed with the CellRanger (3.0.1) pipeline with default settings with the reference mentioned above. For TCR analysis, only cells with at least one TCR alpha chain (TRA) and one TCR beta chain (TRB) were considered as detection TCR. Moreover, each unique TRA–TRB pair was defined as a clone type. The following analyses were based on cells with detected TCR. To analyses the clonal abundance and diversity of cells from each stage, we use the alakazam (1.0.2)^55^ package. For the clonal abundance curve, the 95% confidence interval was estimated via bootstrapping. For the diversity curve, special cases of the generalized diversity index correspond to the most popular diversity measures in ecology. At *q* = 0, different clones weigh equally, regardless of sample size. As the parameter *q* increase from 0 to +∞, the diversity index (*D*) depends less on rare clones and more on common (abundant) ones. For BCR analysis, only cells with at least one heavy chain (IGH) and one light chain (IGK or IGL) were considered high-quality BCR and kept for further analysis. Furthermore, each unique IGH-IGK/IGL pair was defined as a clone type. A clone type was considered clonal if it is detected in more than one cell. iSMART^56^ was used to perform local alignment on CDR3 sequences for T cells and B cells separately, and then those CDR3 sequences were clustered into antigen-specific groups. We selected the top2 abundant CDR3 clusters across conditions, and we calculated the proportions of patients for each CDR3 sequence. Clustal Omega^57^ was used to align CDR3 sequences and generated a guide tree. All plots were generated using ggplot2 (3.3.1),^58^ and heatmaps were generated using pheatmap (1.0.12) unless otherwise specified.

### Statistical analysis

The two-tailed Kruskal–Wallis test, followed bu Dunn’s post-test was used for multiple group comparisons. Because the data in this has a small sample size with large intrapatient variances contributed from both patient-specific effect and sampling time, which is hard to align for asymptomatic patients, therefore the samples were treated as independent. The specific statistical tests and their resultant significance levels are also noted in each figure legend. The R packages Seurat, ggplot2 (version 3.1.0) (Wickham, 2016), GraphPad Prism, and Adobe Illustrator were used to generate figures. P-values were added to the plot by *stat_compare_means* function in ggpubr (0.3.0) package.

## Supplementary information

Supplementary Fig and Table

Data S1

Data S2

Data S3

## Data Availability

All Raw and processed data are available on CNGB Nucleotide Sequence Archive (CNSA) with accession number CNP0001250.

## Code availability

The codes supporting the current study are available from Github (https://github.com/YOU-k/covid_analysis).
