# Supplementary material for: Single-cell immune profiling reveals distinct immune response in asymptomatic COVID-19 patients
Source: Signal Transduct Target Ther. 2021 Sep 16;6:342. doi: 10.1038/s41392-021-00753-7 (PMC8443960; doi:10.1038/s41392-021-00753-7)
Supplement: Supplementary file 1 — Supplementary Fig and Table [file 41392_2021_753_MOESM1_ESM.docx]

Supplementary Materials for

Longitudinal single-cell immune profiling reveals distinct innate immune response in asymptomatic COVID-19 patients

Xiang-Na Zhao, Yue You, Xiao-Ming Cui, Hui-Xia Gao, Guo-Lin Wang, Sheng-Bo Zhang, Lin Yao, Li-Jun Duan, Ka-Li Zhu, Yu-Ling Wang, Li Li, Jian-Hua Lu, Hai-Bing Wang, Jing-Fang Fan, Huan-Wei Zheng, Er-Hei Dai, Lu-Yi Tian, Mai-Juan Ma

Correspondence to: Er-Hei Dai ([daieh2008@126.com](mailto:daieh2008@126.com)), Lu-Yi Tian ([tian.l@wehi.edu.au](mailto:tian.l@wehi.edu.au)), or Mai-Juan Ma ([mjma@163.com](mailto:mjma@163.com))

**This PDF file includes:**

Figures. S1 to S7

Tables S1

Captions for Data S1 to S3

**Figure. S1.**

**Overview of data integration and cell composition for each condition and each sample**. **a**, UMAP plot of cells in each condition. Doublets are not included. Colors represent different cell types in the same way as Fig. 1c. **b**, Bar plot of cell-type percentages for each sample. The x-axis shows the information on patients and days post symptom onset. For the asymptomatic samples, the days are time after the SARS-CoV-2 test positive. Colors represent different cell types in the same way as Fig. 1c and Fig. 2a. **c**, Dot plots of average expression and percentage of expressed cells of marker genes in each labeled cell type.

**
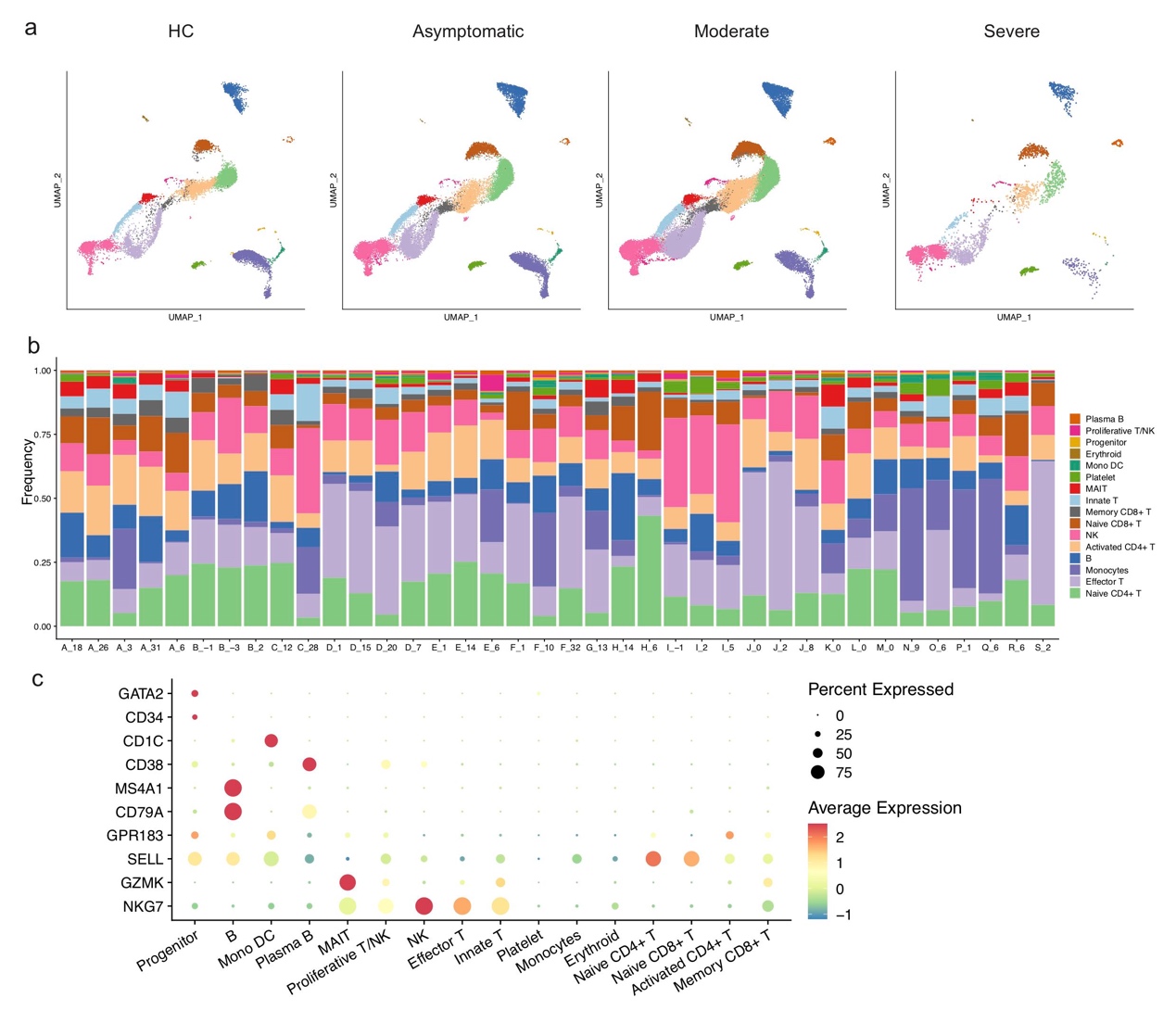
**

**Figure. S2.**

**Visualization of proportions of immune subsets for each condition and time.** **a**, Bar plot of percentage of cell types for each condition and stage. Moderate patient samples were divided into three stages based on days after symptom onset. **b**, Line chart of the proportion of cell types for each sample, samples from the same patient were connected.

**
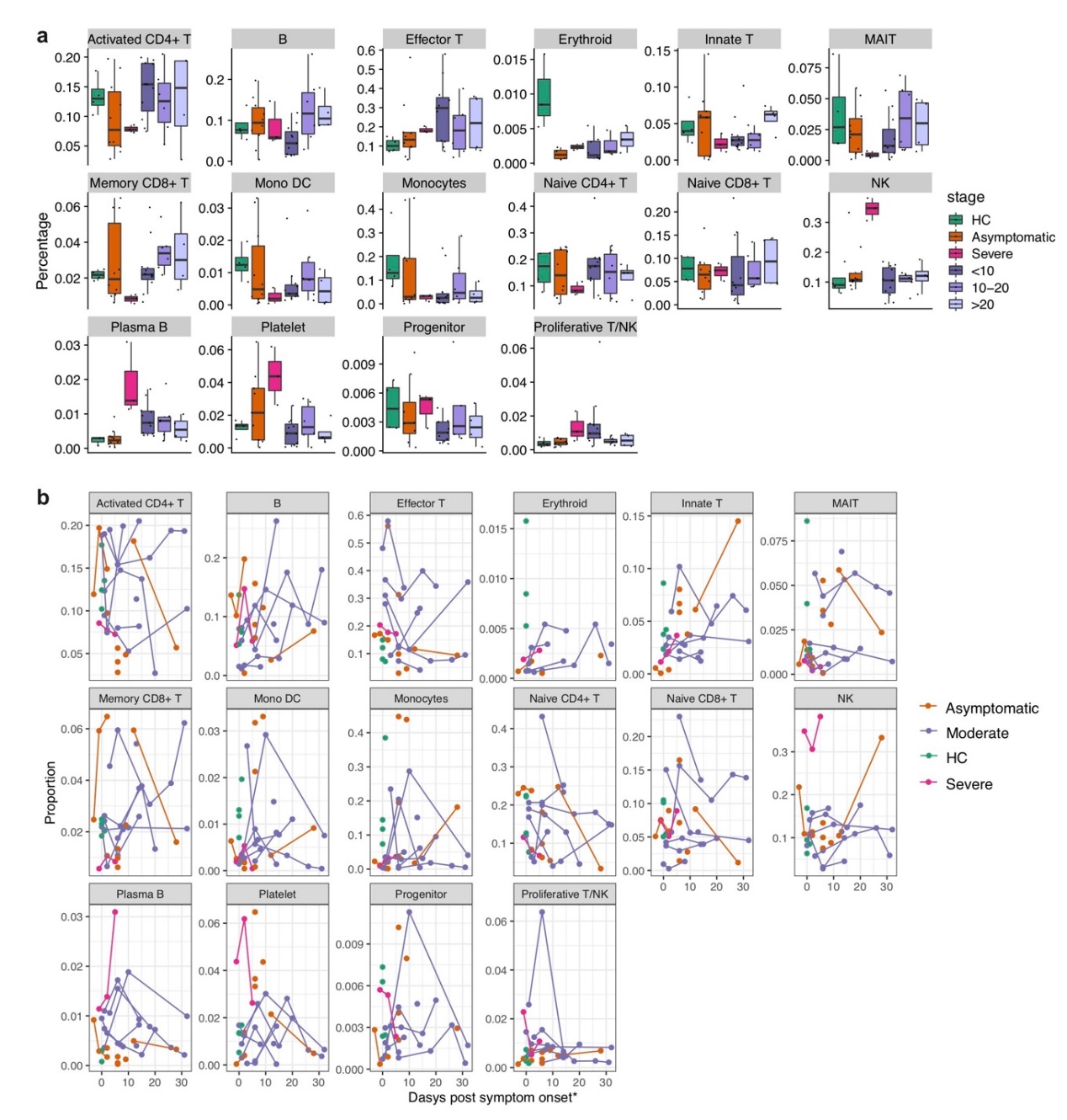
**

**Figure. S3.**

**Overview of T/NK subsets**. **a**, Heatmap of the relationship between clusters generated from all cells (Fig. 1) and clusters generated from T/NK subsets (Fig. 3).

**
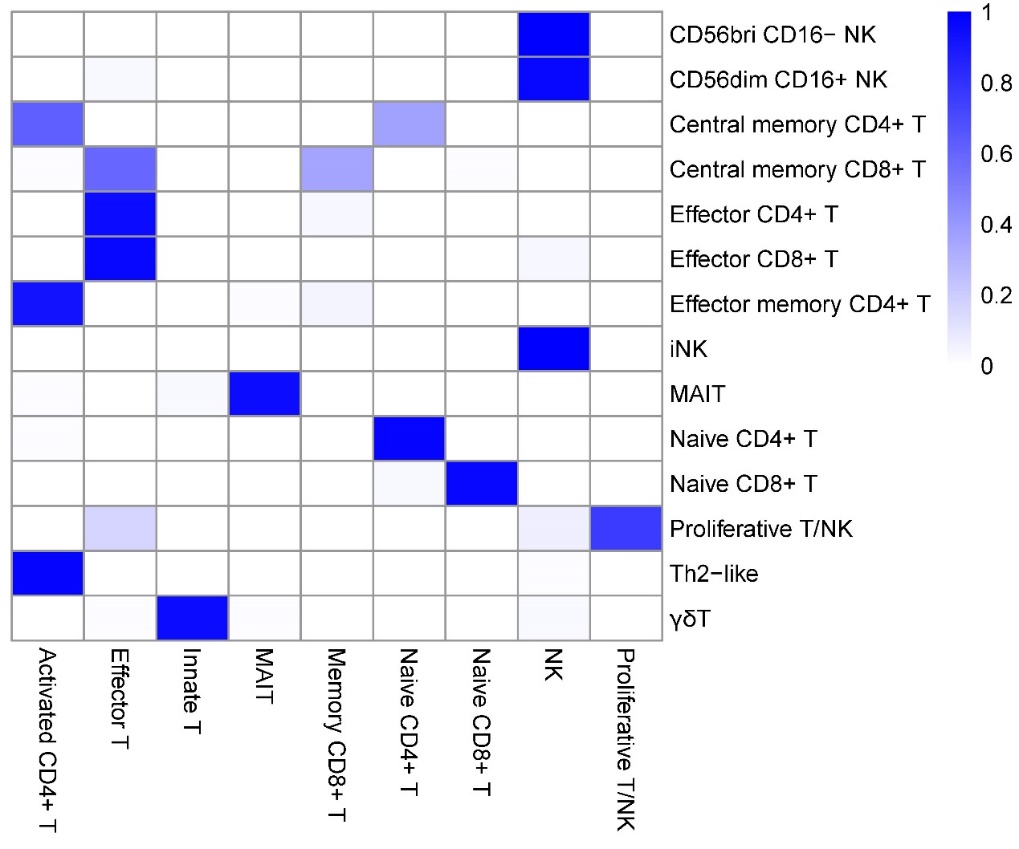
**

**Figure. S4.**

**Visualization of marker gene expression and proportions of cell types across conditions**. **a**, UMAP plots of T/NK subsets, colored by selected markers. **b**, Bar plot of cell-type proportions of each sample in each condition and stage.

**
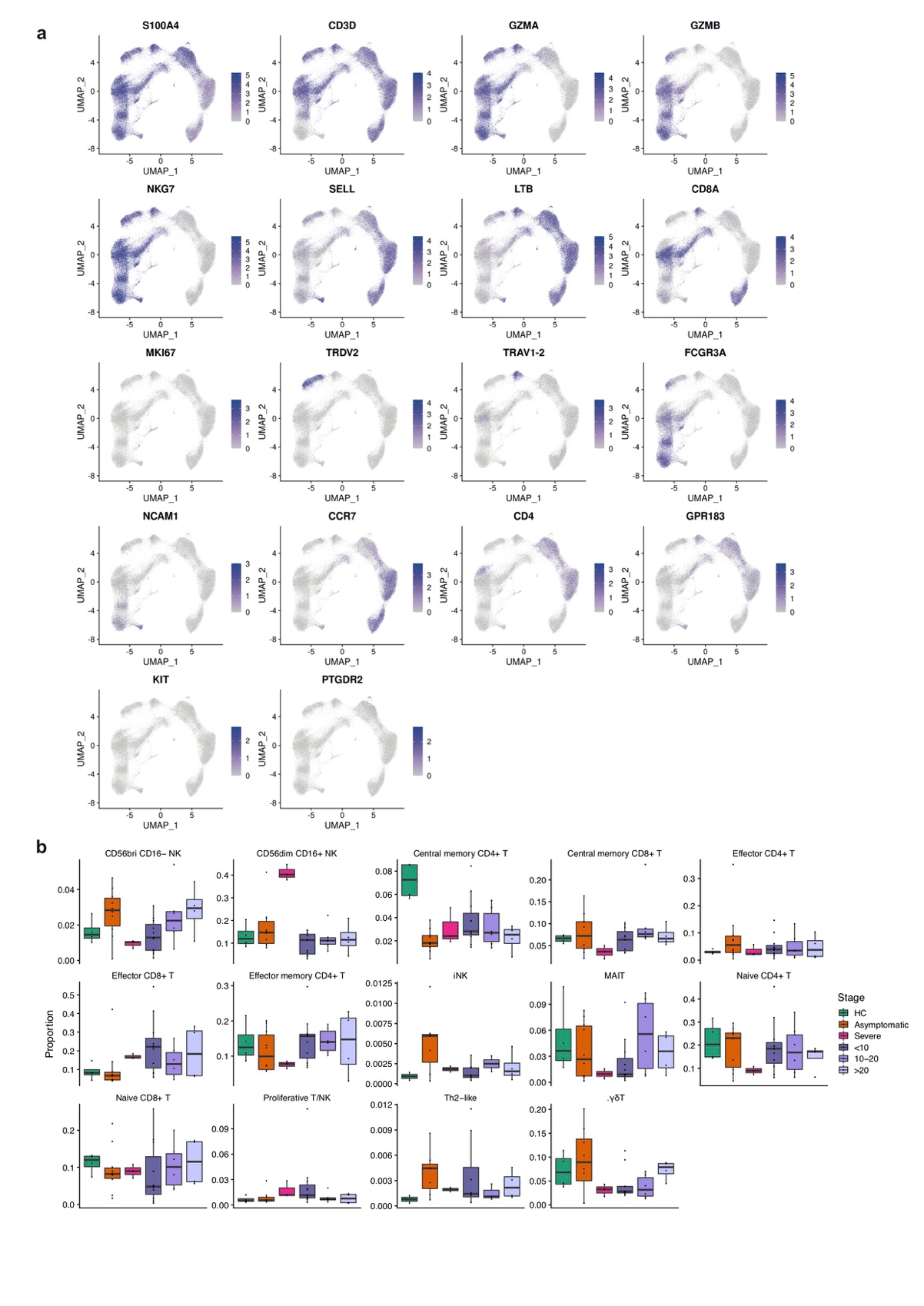
**

**Figure. S5.**

**Cell annotation and marker-gene expression of Th2-like cells**. **a**, SingleR annotation of Th2-like cells with MonacoImmuneData used as the reference. Color represents the number of cells predicted in each reference cell type. **b**, UMAP of all cells (left) and T/NK subsets (right), colored by expression of TNFRSF19. **c**, t-SNE of cells in human lung atlas, colored by four major cell types (left), expression of TNFRSF19 (middle) and ACE2 (right), labeled with cell types that have positive gene expression of these genes.

**Figure. S6.**

**Representative results of differential gene expression analysis and GO enrichment analysis of T/NK subsets.** **a**, Heatmap of selected DEGs in Effector CD8^+^ T cells. **b**, Heatmap of selected DEGs in Effector CD4^+^ T cells. **c**, Heatmap of selected DEGs in CD56^dim^ CD16^+^ NK cells. **e**, Heatmap of selected DEGs in CD56^bri^ CD16^‒^ NK cells.

**Figure S7.**

**Score of IFN-I signaling pathway in CD56^bri^CD16^−^ NK cells, CD56^dim^CD16^+^ NK cells and effector T cells.**

**a** Box plots of the average expression level of IFN-I related genes calculated with AddModuleScore across patients on CD56^bri^CD16^−^ NK cells, CD56^dim^CD16^+^ NK cells, and effector T cells separately. Colors denote disease conditions. **b** Boxplots of the average expression level of IFN-I related genes on CD56^bri^CD16^−^ NK cells, CD56^dim^CD16^+^ NK cells, and effector T cells separately. Boxes are facet by patients and colored by stages.


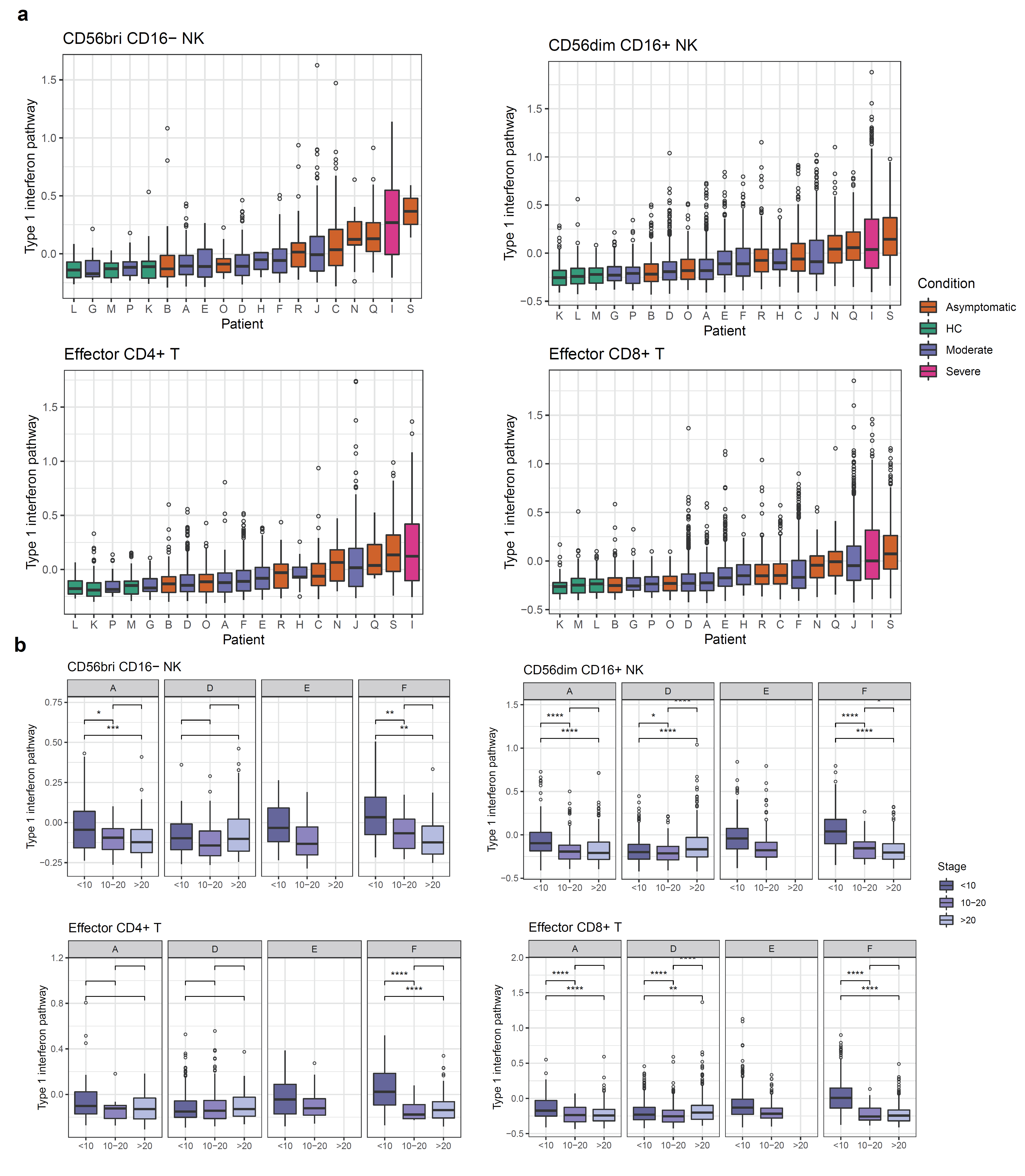


**Figure. S8.**

**Summary of TCR clonal expansion and biased usage of V(D)J genes. a**, UMAP of all T/NK cells. Color represents whether TCR is detected. **b**, Heatmap of proportions of CDR3 sequences obtained in top 2 CDR3 clusters across conditions in T cells. The guide tree is colored by CDR3 clusters. Color of dots represent conditions, and size represent frequency. **c**, Usage of TRBV genes across different conditions and stages. Wilcoxon signed-rank test is used. **d**, Usage of TRBJ genes across different conditions and stages. Two-sided Kruskal–Wallis test was used. **e**, Heatmaps of the difference in TRA/B rearrangement in four conditions. The colors represent the percentage of V-J gene usage.

**Figure. S9.**

**Summary of usage of V(D)J genes and DGE analysis of B cells. a,** Heatmap of proportions of CDR3 sequences obtained in top 2 CDR3 clusters across conditions in B cells. **b**, Heatmaps of the difference in IGH/K/L rearrangement in four conditions (HC, asymptomatic, moderate, and severe). **c**, Heatmaps of selected DEGs in three B cell subsets.

**Table S1.**

**Demographic, clinical, and laboratory characteristics of Covid-19 patients on hospital admission and healthy controls**

**Captions for Data S1 to S3**

Data S1. Differentially expressed genes across conditions on T cells and NK cells.

Data S2. Enriched gene sets of genes with different expression patterns on effector T cells and NK cells.

Data S3. Enriched GO terms on differentially expressed genes in T cells and NK cells
